# Quiescent, Slow-Cycling Stem Cell Populations in Cancer: A Review of the Evidence and Discussion of Significance

**DOI:** 10.1155/2011/396076

**Published:** 2010-09-29

**Authors:** Nathan Moore, Stephen Lyle

**Affiliations:** Department of Cancer Biology, University of Massachusetts Medical School, 364 Plantation Street-LRB 411, Worcester, MA 01605, USA

## Abstract

Long-lived cancer stem cells (CSCs) with indefinite proliferative potential have been identified in multiple epithelial cancer types. These cells are likely derived from transformed adult stem cells and are thought to share many characteristics with their parental population, including a quiescent slow-cycling phenotype. Various label-retaining techniques have been used to identify normal slow cycling adult stem cell populations and offer a unique methodology to functionally identify and isolate cancer stem cells. The quiescent nature of CSCs represents an inherent mechanism that at least partially explains chemotherapy resistance and recurrence in posttherapy cancer patients. Isolating and understanding the cell cycle regulatory mechanisms of quiescent cancer cells will be a key component to creation of future therapies that better target CSCs and totally eradicate tumors. Here we review the evidence for quiescent CSC populations and explore potential cell cycle regulators that may serve as future targets for elimination of these cells.

## 1. Cancer Induction from Adult Stem Cells

The development of cancer is a complex multistep process that requires the accumulation of mutations resulting in a cell acquiring the essential hallmarks of cancer: evasion of apoptosis, self-sufficiency in growth signals, insensitivity to antigrowth signals, invasive and metastatic abilities, limitless replicative potential, and sustained angiogenesis [1]. Given that normal adult stem cells already exhibit limitless replicative potential, it is hypothesized that transformed stems cells may be the cells of origin for many cancers [2, 3]. In addition to replicative potential, long-lived stem cells have the opportunity to accumulate oncogenic mutations over years or decades from common mutagenic sources like inflammation, radiation, chemicals, or infection, unlike shorter-lived transit amplifying (TA) cells that rapidly proliferate and differentiate [4, 5]. Like healthy adult stem cells, transformed stem cells are expected to be able to generate oncogenic TA cells. These TA cells would be capable of driving tumor formation and generating the heterogeneous combination of populations commonly seen in cancer [6, 7]. Transformed stem cells have been termed cancer stem cells (CSCs), also known as cancer initiating cells, and are defined as the fraction of cells within a tumor that are long lived, possess the potential to proliferate indefinitely, and can generate all heterogeneous lineages of the original tumor in xenograft models [6, 8]. CSCs are expected to utilize characteristics commonly found in stem cell populations such as differential metabolic activity, specific signaling pathway activity, and regulation of cell cycling characteristics, albeit with aberrant regulation [7, 9] (Table 1). Importantly, CSCs that survive treatment could account for tumor recurrence as a result of reactivation of proliferation in surviving CSCs [10]. Traditional chemotherapy regimens target proliferating cells, potentially missing slower dividing CSCs that must be eradicated to provide long-term disease-free survival [11]. A better understanding of CSCs is essential in understanding the biological and clinical consequences of existing regimens and designing new therapies to improve patient outcome [9]. 

Current methods for isolation and study of CSCs rely on cell surface markers found to be enriched in populations with stem cell-like properties. This technique was first used by Bonnet and Dick in 1997 when they demonstrated that only the CD34^+^CD38^−^ subset of cells were capable of initiating human acute myeloid leukemia (AML) in immune-compromised mouse models [12]. Since the work of these two pioneers, CSC populations have been identified in multiple epithelial cancers including the breast [8], prostate [13], pancreas [14], colon [15–17], ovaries [18], and brain [19]. 

Unfortunately, the use of CSC markers has not been without controversy. One issue centers on uncertainty of the functional implications of CSC markers and is best exemplified by the use of CD133 in the identification of colon CSCs. Shortly after Ricci-Vitiani et al. (2007) demonstrated the use of the CD133 to identify CSCs in colon tumor, Shmelkov et al. demonstrated that CD133 expression was not restricted to colon CSCs, but that CD133 is expressed on differentiated colonic epithelium in both mice and humans [16, 20]. Reasons behind this contradiction of data still remain unclear, but Kemper et al. methodically evaluated the CD133 antibodies used by both groups and reached the conclusion that the AC133 epitope used by Ricci-Vitiani et al. recognized a differentially expressed form of CD133 that is not recognized by the antibody used by Shmelkov et al. [21]. It appears that CD133 is expressed in all colon epithelium, while the AC133 epitope is specific for the CSC phenotype. Furthermore, Kemper et al. were unable to determine the functional significance of the differentially expressed isoforms of CD133, highlighting another drawback to the use of markers to identify CSCs. Very little is known about the function of many of the proposed CSC markers, and even less is known about the functional implications they may have for the CSC phenotype. At best, markers without functional implications must be viewed as only tools for stem cell enrichment, suggesting the need for a more functionally significant means of CSC identification [22]. Given the similarities between normal adult stem cells and CSCs, aberrant regulation of self-renewal and quiescence is likely central to CSC pathology [9, 10]. Targeting pathways that mediate stem cell quiescence is therefore an intriguing alternate method for CSC identification and use in future therapy.

The primary objectives of this paper are to place quiescent label-retaining studies in the context of what is currently known about adult stem cells and then review the existing evidence for quiescence in cancer stem cells. We will examine current evidence for the role of quiescence in CSC resistance to conventional cancer therapy and recurrence. Finally, we will explore current knowledge of quiescence regulation and how these studies might be considered when developing CSC future experiments to develop targeted therapies against CSCs.

## 2. Adult Stem Cells and Quiescence

Adult stem cells are critical for continued normal tissue homeostasis and response to wounding for many of the epithelial tissues of the body. Adult stem cells are characterized by their ability to self-renew indefinitely and produce progeny capable of differentiating and repopulating tissue specific lineages [7]. Populations of adult stem cells have been identified in tissues throughout the body, including the skin [23–25], mammary glands [26, 27], intestine [28, 29], prostate [30], brain [31], and the hematopoietic system [32, 33]. In tissues where cells are frequently lost to the environment, like those of the intestine and skin, new cells are continuously required to replenish those that are lost. To facilitate this constant need for new cells, some epithelial tissues are arranged hierarchically with slowly proliferating stem cells that asymmetrically divide to give rise to a new stem cell and a rapidly dividing transit amplifying (TA) cell [34]. Transit amplifying cells proliferate quickly for a limited number of divisions, allowing for the high degree of cell turnover necessary to sustain adult tissues. Infrequent division or a quiescent nature is not definitive for adult stem cell but is suggested to be important for maintenance of many adult stem cell pools. Evidence suggests that quiescence may play an important role in protecting stem cells from exhausting their proliferative capacity, inhibiting differentiation, and limiting accumulation of mutations during frequent rounds of DNA synthesis [35–37]. 

**Figure 1:**
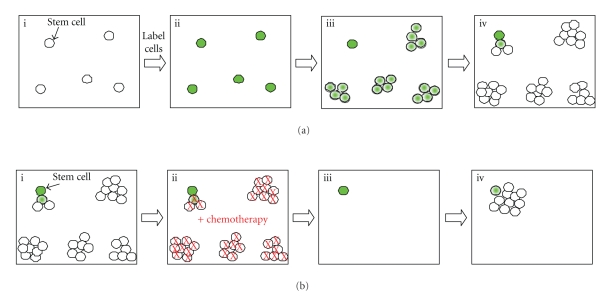
*Pulse Chase Labeling and Chemotherapy Survival of Stem Cells.* (a) Cell suspensions are labeled with BrdU or other label ((i) and (ii)). As rapidly proliferating transit amplifying cells divide, label is diluted among the daughter cells and eventually becomes undetectable (iii). Slow dividing stem cells retain label occasionally producing a new transit amplifying cell that will quickly dilute out residual label (iv). (b) Heterogeneous tumors are predicted to contain a population of slow cycling label retaining cells (i). Conventional chemotherapies target and kill rapidly proliferating cells, while quiescent cells survive ((ii) and (iii)). Cancer stem cells that survive chemotherapy re-enter the cell cycle and re-establish the tumor.

Initial efforts to identify and study adult stem cells took advantage of the slow-cycling nature of stem cell populations in studies employing pulse/chase methodology [23, 28]. In these studies, tritiated thymidine (^3^H-TdR) or 5-bromo-2-deoxy-uridine (BrdU) was repeatedly administered to mice or cultured cells that were then followed by an extended period of chase time. During this chase period, rapidly proliferating TA cells divide the label between daughter cells, consequently diluting the label (Figure 1). In contrast, slow-cycling stem cells undergo few divisions and retain detectable quantities of label for much longer periods of time. Cotsarelis et al. demonstrated that label retaining cells (LRCs) were exclusively present in the bulge area of the mouse hair follicle [23]. These cells were found to be relatively stem-like: “primitive” in cytoplasmic contents, structurally similar to other putative stem cell populations, and could be stimulated to proliferate. We utilized human skin xenografted onto immunodeficient mice to show that LRCs were present in an analogous bulge region of human skin delineated by keratin 15 (K15) expression [24]. Cells present in the bulge region have been experimentally shown to be quiescent for up to 1 year [38], and based on the hair growth cycle of scalp skin can likely remain quiescent for up to 5 years. Using the K15 promoter to drive expression of EGFP or *lacZ*, K15 positive cells were found to differentiate into all major epithelial lineages of the mouse skin [39]. We demonstrated that K15+ bulge cells from human skin can differentiate into epidermal, sebaceous, and hair follicle lineages *in vitro* [40]. Array analysis of the LRC bulge population showed increased activation of Smad and inhibitors of the Wnt pathway, suggesting the ability for LRCs to organize their niche and communicate with neighboring mesenchymal and epithelial cells, an important characteristic for stem cell function [41]. 

The work in our lab and others supports a model in which the bulge region of hair follicles represents the stem cell niche in skin. At the onset of the growth phase (anagen) hair follicle stem cells are activated and produce matrix TA cells that proliferate and differentiate into the seven different lineages found within the hair follicle. As matrix TA cells exhaust their proliferative potential they enter a state of destruction (catagen) leading to the loss of the majority of the hair follicle *excluding* the bulge. Catagen is followed by a period of rest (telogen) in which the bulge stem cells remain quiescent until activation into a new anagen stage [2].

Although likely important for the maintenance of the stem cell pool, quiescence may not be a requirement for adult stem cells. Using a *lacZ* construct under a conditional promoter for the stem cell-associated protein leucine-rich G protein-coupled receptor 5 (Lgr-5), Jaks et al. demonstrated a distinct nonlabel retaining subpopulation of bulge cells that overlap with the CD34^+^/K15^+^ at telogen but not anagen [42]. Lineage tracing techniques confirmed that Lgr-5^+^ cells actively cycled during normal homeostasis and had a multipotent phenotype. The authors of this paper suggest that the Lgr-5^+^ population of cells represents a cycling population of stem cells under normal conditions, whereas the label retaining CD34^+^/K15^+^ stem cells may represent a reserve population that is activated after tissue damage. As yet, a conclusive relationship between these two populations cannot be firmly established. 

Similar label-retaining methods have been used to study slow-cycling cells in other tissues, such as the small intestine and colon. Work conducted by Potten and colleagues identified slow-cycling LRCs at the +4 position at the base of the colon crypt. These crypt base cells were found to be maintained in a steady state of between four and six cells that go through division approximately once a week [43]. Upon irradiation, these cells demonstrated increased antiapoptotic bcl-2 expression, decreased p53 expression and were highly activated and involved in clonogenic regeneration of the crypt. Detailed biochemical analysis of this population has been limited by the absence of reliable markers and methods capable of sufficiently isolating these cells. Two studies involving the putative stem cell-associated RNA binding protein Musashi-1 (Msi-1) have both demonstrated colocalization of this protein with colon LRCs, but fell short of testing for clonogenicity of this population [44, 45]. Similar to stem cell populations in the skin, *β*1-integrin was found to be highly expressed in the lower half of the colonic crypt [46]. When sorted via flow cytometry, *β*1-integrin showed enrichment for clonogenic cells; however, an exact colocalization pattern with LRCs was not evaluated, and therefore, the connection remains only speculative. 

From the evidence collected in these studies and others, a model has been suggested in which slow-cycling stem cells, found at the base of the crypt, undergo periodic division to give rise to TA cells. Transit amplifying cells low in the crypt undergo rapid division and slowly progress up the crypt, losing replicitative potential and differentiating as they increase in crypt height. These cells are ultimately lost to the environment [6, 43]. 

As within the hair follicle, there is convincing evidence for an Lgr-5^+^ nonlabel retaining population of colon stem cells additionally found at the base of the crypt [29]. While the LRCs reside at the +4 population, Lgr-5 cells are observed as slender wedge-shaped cells at the +2 position. Again, the exact relationship between the LRCs and the Lgr-5^+^ cells is yet to be fully explored, and more data into the lineage potential of both of these cell populations is needed to form a cohesive model. 

Since the early identification of colon and hair follicle slow-cycling stem cell populations, label-retaining techniques have been used to identify and validate putative stem cell populations in multiple epithelial tissues. In the mammary gland, three separate label-retaining populations have been identified and proposed as possible stem cells. In a study conducted by Welm et al., LRCs were found to comprise a subpopulation of stem cell antigen-1 positive (Sca-1^+^) cells [26]. These Sca-1^+^ cells were found to be enriched for the ability to form outgrowths, leading the authors to speculate that the LRCs might represent the stem cell population contained within the Sca-1^+^ cells. In contrast to this study, Shackleton et al. identified a long-term label-retaining population enriched by the marker combination Lin^−^CD29^hi^CD24^+^ that was able to reconstitute a functional mammary gland from a single cell [27]. The Lin^−^CD29^hi^CD24^+^ did not enrich for the Sca-1 population, prompting other groups to suggest a stem cell hierarchy in which multiple layers of stem cells exist within the mammary gland [47]. Using a slightly different methodology, Pece used the lipophilic fluorescent dye PKH26 to identify a population of mammary label retaining cells [48]. The use of the PKH26 allows for live sorting of LRCs, which is not possible using the nucleotide analogue BrdU and ^3^HT-TdR that both require permeabilization of the cell membrane for antibody labeling. Live sorting of PKH26 LRCs demonstrated increased *in vitro* sphere formation efficiency and regeneration of cleared fat pads over non-LRCs. Pece was also able to conduct transcriptional analysis of the LRC population, from which he created a human normal mammary gland stem cell signature (hNMSC) consisting of the markers CD49F/DNER/DLL1. Unfortunately, the exact relationship between the different populations identified by these three groups is not yet clear.

In the brain, high doses of ^3^H-TdR kill all but one percent of proliferating subependymal. High dose therapeutics did not affect the capacity of quiescent cells to generate spheres *in vitro* or repopulate the proliferating population *in vivo*. The ability to survive and re-enter the cell cycle suggests a stem cell phenotype for these quiescent cells [49]. Prostate slow-cycling LRCs located in the proximal ducts demonstrated high proliferative potential and the ability to reconstitute the prostate glandular structure *in vitro.* This ability singles them out as stem cells over more rapidly cycling TA cells located at the distal region of the ducts [50]. Finally in the pancreas, characterization of LRCs around the acini and ducts suggested a stem cell population by demonstrating increased expression of the putative stem cell marker c-Met and activation in response to damage to form duct-like structures [51]. 

Combined, these data indicate an important role for quiescent LRCs in maintenance and longevity of multiple adult epithelial tissues.

## 3. Quiescence and CSCs

**Figure 2:**
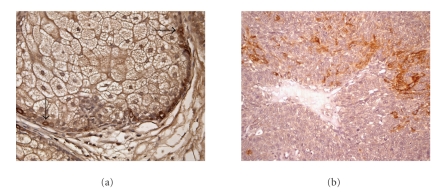
*Stem cell markers in Normal Sebaceous Gland and Sebaceous Tumor.* Immunohistochemical staining for the skin stem cell marker Keratin 15 (K15). (a) Normal skin sebaceous gland with labeled stem cells (black arrows). (b) Sebaceous tumor with heterogeneous expression of K15.

If CSCs do originate from normal adult stem cells, then it is foreseeable that key stem cell regulatory traits are retained through the oncogenic transition; quiescence is potentially one of these traits. Little research has been done to address how quiescence might play a role in CSC biology, but there are some indications that quiescent stem-like populations might contribute to at least some tumors. We previously identified a subpopulation of cells in human sebaceous tumors that expressed the skin stem cell marker keratin 15 [52] (Figure 2). These cells appeared to have variable expression of the proliferation marker Ki-67, suggesting a low but higher proliferative rate than normal stem cells. In primary ovarian tumors, Gao et al. demonstrated that CD24^+^ cells expressing stem cell-associated genes like *nestin, oct4*, and both *notch1* and *notch4* were more slowly proliferating than the bulk tumor cells suggesting a quiescent phenotype [18]. Low numbers of slowly proliferating CD24^+^ cells were shown to produce tumors in a xenograft model where bulk cells were found to be nontumorigenic. This data implicates a link between quiescence and ovarian tumor CSCs.

Pece also observed a link between CSCs and quiescence in breast tumors [48]. Using the hNMSC signature generated with normal mammary LRCs, Pece turned his attention to the analysis of primary breast tumors, finding that the hNMSC signature was more commonly found in grade 3 tumors over that of grade 1. When grade 1 and grade 3 mammospheres were analyzed for PKH label retaining cells, both populations were found to retain label, with grade 3 tumors demonstrating a higher percentage. This data suggests an increase in stem-like cells as tumors progress. When evaluated for tumor genicity, breast tumor cells positive for the hNMSC signature were more efficient at forming *in vitro* spheres and *in vivo* xenograft tumors that those cells lacking the hNMSC signature. 

Cultured cancer cell lines are often used to study signaling pathways, invasion, migration, and apoptosis, but are rarely thought of as candidates for CSC studies. Many of the most widely used cell lines have been in passage for years, are perceived homogeneous, lack interactions with the appropriate stromal microenvironment, and change characteristics based on alterations in culture conditions. Therefore, cultured cell line studies assessing CSC characteristics must be evaluated critically, with data interpreted within the context of the experimental parameters, and results confirmed under biologically relevant conditions. Still, interesting work in the cultured tumor lines MCF10A, MCF7, SUM149, SUM159, SUM1315, and MDA.MB.231 suggests that these lines may not be as homogenous and void of “stem like” cells as once thought [53]. CD44^+^/CD24^−^/ESA^+^ cells within these lines were found to contain the ability to self-renew, reconstitute the parental line, and to be up to 90% label retaining. If LRCs are found to retain the CSC phenotype in cultured cell lines, these cell lines may provide an important resource for future delineation of quiescent pathway regulators.

Additional transitive evidence linking quiescence to CSCs can be found in the work conducted by Roesch et al. in melanoma [54]. This group found that primary melanoma cell lines contained a PKH26 label retaining population that was almost specifically identified by the H3K4 demethylase JARID1B. This population of cells was found to incorporate BrdU more slowly but retain it for a longer period of time, lack Ki67 staining, and have a doubling time of up to 4 weeks *in vitro*. When EGFP was placed under the control of the JARID1B promoter, GFP^+^ cells demonstrated increased sphere forming ability *in vitro*. Interestingly, GFP^+^ cells were able to retain BrdU *in vivo*, but did not show increased tumor initiating ability over GFP^−^ cell during the time period analyzed. Small hairpin RNA (shRNA) knockdown of JARID1B resulted in the *in vitro* exhaustion of proliferating cells, demonstrating the need for JARID1B cells in maintenance of proliferative capacity but not initiation of tumors. When assessed more fully, both *in vitro* and *in vivo* GFP^−^ cells gave rise to heterogeneous progeny, including JARID1B GFP+ cells.

The most direct evidence to date for quiescence playing a role in CSCs comes from a study conducted by Dembinski and Krauss [55]. In this study Vybrant DiI cell-labeling solution was used to label pancreatic adenocarcinoma cells and conduct cancer stem studies on flow cytometry sorted label retaining cells. DiI label retaining slow-cycling cells (DiI^+^/SCCs) comprised ~3% of total cell number. Interestingly, label retaining cells also exhibited an elongated fibroblast shape and an increase in the epithelial-mesenchymal transition markers vimentin, snail, and twist. A fibroblast-like CSC is consistent with evidence demonstrating an increase in stem-like properties in cells that have undergone an epithelial-mesenchymal transition [56]. Furthermore, sorted DiI^+^/SCCs demonstrated a 2.5–10-fold increase in soft agar colony forming ability, twofold increase in invasive potential, and more than a tenfold increase in xenograft formation over nonlabel retaining cells. Combined, these data suggest that DiI^+^/SCCs cells represent an enriched CSC population. When assessed for common CSC marker status, DiI^+^/SCCs were enriched but only partially overlapped with CD24^+^/CD44^+^ and CD133^+^ populations. It is curious to consider how these commonly used CSC markers relate to the LRC populations and what role, if any, these markers play in the slow-cycling phenotype? 

Like the melanoma study by Roesch et al. [54], Dembinski and Krauss's study also indicated the ability for LRCs to produce non-LRCs and surprisingly also for non-LRCs to produce LRCs. Such a dynamic suggests two possibilities (1) that the true unknown CSC population is favored in the LRCs, but also found in the non-LRCs and can therefore give rise to both populations, or (2) that there exists a dynamic relationship in LRC-CSC populations that is context dependent and allows for interconversion between the two states. The Dembinski and Krauss study argues a dynamic population of CSCs that might coincide with an epithelial-mesenchymal transition (EMT). EMT plays a central role in embryogenesis and mesoderm differentiation into multiple tissue types during development [56]. The emergence of embryonic stem cell-associated genes like *nanog, oct4, sox2*, and *c-myc* in high grade undifferentiated cancers is suggestive that aberrant regulations of EMT and other early development pathways might be playing a role in CSC characteristics [57]. This data is a further evidence to support a dynamic quiescent slow-cycling model for many types of cancer. Future studies will be important for further development and integration of these observations into the CSC model for tumor initiation and propagation.

## 4. Quiescence and Resistance to Chemotherapy

At the present time, we have no clear understanding of why some patients recur and which cancers will have resistance to conventional types of therapy. Tumors from different patients in the same organ are likely to have undergone different oncogenic transitions, leading to a diversity of possible regulatory mechanism and pathway activities that might be contributing to the survival of a specific cancer. While broad patterns like the dysregulation of the Wnt pathway in colon carcinomas are commonly observed, the secondary mutations that may accompany these cancers could be vastly different and contribute to survival in different ways [58]. Even within the same tumor, different CSCs have the possibility to accumulate unique mutations that may provide added resistance and be passed on to daughter cells. In context with the vast differences in tumorigenesis and heterogeneity with a tumor, it is not surprising that the exact contributors to chemotherapy resistance and consequently which patients will respond optimally to chemotherapy are not well understood. It has been proposed that variations in cell cycle control, antiapoptotic proteins, increased DNA damage repair proteins, upregulation of cellular pumps, and increased metabolic activity may all play important roles in chemotherapy resistance [6, 59–62]. 

Conventional chemotherapies and radiotherapies target proliferating cells and require active cycling for induction of apoptosis. The quiescent nature of many adult stem cell pools is therefore an inherent mechanism for resistance and cell survival to conventional therapies. In the hematopoietic system, normal hematopoietic stem cells (HSCs) contain high levels of the quiescence regulator p21^cip1/waf1^ (p21) [63]. When treated with the commonly used chemotherapy agent 5-fluorouracil (5-FU), mice that were p21 deficient had a significant decrease in cobblestone area-forming stem cells (10.8%) than normal p21 expressing wild-type mice (60.5%). In the brain, Morshead et al. demonstrated that high doses of tritiated thymidine (^3^H-TdR) killed the constitutively proliferating cells in the adult mouse forebrain, but had no effect on quiescent stem cell ability to generate spheres [49]. This data supports a model in which quiescent mouse forebrain stem cells are able to survive and re-enter the cell cycle to allow for regeneration of the damaged tissue. A similar pattern of stem cell survival and regeneration was observed 72 hours following doxorubicin treatment in mouse intestine. In this experiment, mice intestine demonstrated increased amounts of cell death via apoptosis in the +3–6 positions and a parallel disappearance of mitotic activity [64]. This period of relatively nonexistent mitotic activity was followed by stem cell re-entry into the cell cycle and tissue regeneration in the +4 position stem cell compartment. Furthermore, colon stem cell survival during chemotherapy is aided by increased expression of BH3-only bcl-2 members that inhibit apoptosis, working in parallel with quiescence to increase the likelihood of stem cell survival [65]. In chemotherapy-induced alopecia, the rapidly dividing TA cells in the hair matrix undergo apoptosis, while the stem cells in the bulge region survive to regenerate the follicle after chemotherapy is withdrawn. Potential factors involved in regulating hair follicle stem cell survival such as caveolin-1 are emerging [66]. 

Similar mechanisms for survival and self-renewal for CSCs are plausible in instances of tumor recurrence in human patients where cytotoxic agents kill proliferative cancer cells, leaving quiescent slow-cycling [6]. Cancer stem cells that survive chemotherapy would have the ability to re-enter the cell cycle and produce highly proliferative-rapidly dividing progenitor cells that can re-establish the tumor. It is even probable that successive cycles of chemotherapy would intensify a tumor by weakening the normal stem cell pool and creating therapy resistant CSCs that give rise to resistant off-spring [9]. 

Slow cycling CSC populations in the colon, breast, ovaries, and pancreas have been shown to demonstrate both *in vivo* abilities to survive therapies that kill bulk tumor cells as well as a requirement for doses of up to twice that which are required to kill rapidly proliferating cells *in vitro* [18, 55, 62, 67]. These data demonstrate how ineffective conventional therapies can be on quiescent cell populations and help to explain why tumors that seem to fully regress during treatment can recur. While large tumor populations may appear to have totally regressed after treatment, single surviving CSCs would not be detectable with current diagnostic technology. Populations of CSCs that are resistant to chemotherapy or radiation are able to re-enter the cell cycle or never fully undergo cell cycle arrest and are primed to re-establish tumors [53, 68]. Even more devastating to the survival of patients may be CSC response to stress from chemotherapy and radiotherapy. Mouse ovarian tumors have been demonstrated to undergo accelerated clonogenic production during radiotherapy regimens, expanding the CSC pool and driving development of a more aggressive secondary tumor [69]. Furthermore, these cells would be more likely to produce chemotherapy resistant offspring, rendering the tumor unaffected by later rounds of treatment.

While quiescence is likely to contribute to the survival of CSCs in response to chemotherapy and radiation, slow cycling is not the sole mechanism and in all likelihood works in parallel with other systems to increase survival. Msi-1^+^ colon cancer cells have been demonstrated to be less sensitive to cytotoxic drugs due to increased IL-4 expression and orchestration of antiapoptotic mechanisms [70]. The expression of other antiapoptotic proteins like c-Flip and Bcl-2 BH-3 only family members is frequently seen in stem cell and CSC populations and has been demonstrated to contribute to cell survival during radiation and chemotherapy [59, 60]. Reduced cycling may help to limit cell damage in these cases, decreasing prodeath signals and increasing the potential for CSC survival. 

Additional mechanisms for CSC survival include increased DNA damage repair, upregulation of cell pumps like the multidrug resistance transporter (MDR1) and the Adenosine triphosphate-binding cassette (ABCB1), and increased metabolic activity through ALDH [61, 62]. Although the quiescence contribution to these mechanisms of resistance is unclear, it is likely that reduced proliferative rate only adds to their effectiveness. Additional time in S or G_2_ phase of the cell cycle coupled with increased DNA repair protein activity may afford a survival advantage over bulk cells that continuously accrue DNA damage and ultimately are forced to undergo apoptosis. Reduced cycling speed together with increased pumps would facilitate more drug being removed from CSCs, limiting overall cytotoxic effects during the period of treatment. Additionally, quiescence would allow for increased metabolic activity of ALDH and other metabolites over that of bulk cells with a shorter cell cycle period. Importantly, there is no reason why combinations or all of these resistance mechanisms could not be playing a role in CSC survival. Future therapies may need to address all these issues to be successful in complete tumor eradication.

## 5. Regulators of Quiescence

Given the importance of quiescence in the CSC contribution to tumor progression and survival, understanding the mechanisms that govern quiescence will prove important in the development of future strategies to better target these cells. Much of our current understanding of the mechanisms controlling quiescence come from studies using conditional induction of quiescence in normal adult fibroblasts. The induction of quiescence in fibroblasts is generally accomplished in one of three ways: mitogen deprivation, contact inhibition, or loss of adhesion. Each method of inducing quiescence in fibroblast appears to yield a different quiescent transcriptional program [35]. The three transcription programs overlap in differential expression of 131 genes that Coller et al. have designated a “quiescence signature.” This signature is comprised of genes that regulated cell growth and division, suppress apoptosis and differentiation, and govern intercellular communication. Downregulated elements in the quiescence signature consist of genes associated with cell cycle progression including *cyclin B1, cdc20, cul-1, *and* myc*. Up regulated genes included important cell cycle regulators like *TP53 (p53), cyclin D2, *and* MXI1*. Also up regulated in this signature are regulators of key stem cell-associated pathways including the Wnt pathway (*FZD2 *and* TCF7L2*), the BMP pathway (*SMAD1*), and the Notch pathway (*Hes1*). Notch activation of Hes1 is of particular interest as it has been shown to control reversibility of fibroblast quiescence by blocking differentiation and entry in irreversible cell cycle arrest [36]. Notch pathway activity is important in mammary gland development as well as the mammary CSC response immediately following irradiation, suggesting that the Notch pathway may be a potential target in CSCs [5, 71]. 

Interestingly, there exists a fourth transcriptional program in fibroblasts induced by overexpression of cyclin-dependent kinase inhibitors (CKI) like p21 and p16^INK4a^ [35]. The CKI p21 has been found to control entry into quiescence and maintenance of the quiescent state, allowing cells to activate a DNA damage-like response [72]. Additionally, maintenance of fibroblast quiescence has also been shown to be highly regulated by the retinoblastoma family members Rb and p107 [73]. Loss of Rb and p107 did not affect the ability of fibroblasts to enter G_0_, but these cells were unable to maintain the quiescent state. While Rb loss is generally associated with the progression of cancer, retention of Rb in CSCs or contribution of other Rb family members like p107 may be important in CSC maintenance of quiescence.

Developing and studying a quiescence signature in fibroblasts may be important in understanding regulation of the cell cycle, but the exact relevance to quiescent stem cell populations is not very clear. Primarily, quiescence fibroblast studies are conducted on large populations of fibroblasts under biologically stressful conditions like contact inhibition or serum starvation. In contrast, individual stem cells and CSCs maintain quiescence while in contact with daughter cells and stromal layers and in the presence of normal mitogenic signals. Additionally, sphere forming assays commonly used for the identification of stem cells and CSCs rely specifically on proliferation under nonadherent conditions. If mitogen deprivation, loss of adhesion, and contact inhibition truly activate three different transcriptional programs in quiescent fibroblast populations, it is possible that the transcriptional program facilitated by quiescent stem cells and CSCs may be very different. 

Quiescence regulation of a stem cell population is most comprehensively understood in the hematopoietic system. When compared to differentiated or cycling HSCs, quiescent HSCs were found to have up-regulated genes associated with cell cycle regulation, translation and RNA processing, and metabolic process [74]. Down-regulated genes were generally associated with transcription factors, signaling proteins, cell cycle proteins, and inhibitors of cell cycle progression. In line with these findings, the CKI p21 was found to be necessary for quiescence and maintenance of the HSC pool [63]. Mice that are p21 null demonstrate an increase in the number of stem cells present and lose the ability to repopulate the bone marrow in serial transplant experiments, suggesting uncontrolled expansion and eventual exhaustion of the stem cell pool. This deregulation of the stem cell pool is likely due to p21 downstream effects on Rb family members: Rb, p107, and p130. Rb family members play important roles in regulating E2F activity and G_1_/S transition. Triple knockout of these three family members resulted in hematopoietic progenitor G1/S transition and proliferation, leading to exhaustion of the proliferative potential, similar to that seen in p21 loss [37]. 

While p21 also appears to play a role in adult neural stem cell regulation and maintenance, other factors have been shown to be important contributors to quiescent stem cell activation [75]. Occasional exit of neural stem cells from the quiescent state is important for proper tissue maintenance and may be controlled though notch signaling via Hes1 oscillations [76]. Down-regulation of Hes1 in neural progenitor cells during G_1_ phase reduced repression of cyclinD, ngn2, and Dll1, activating Notch signaling and driving cell cycle progression and generation of neural progenitors. Neural progenitors and neurons continue to retain low levels of Hes1 as they proliferate and differentiate. In neural stem cells, Hes1 expression and control of cyclinD and notch signaling increase until subsequent G_1_ entry. Interestingly, p21 loss does not appear to play a significant role during differentiation in the brain, suggesting the need for additional means of cell cycle regulation in differentiated senescent cells [37]. 

Signaling pathways with interactions to other CKIs also play important roles in quiescent adult stem cell regulation. In mammary glands, the Hedgehog pathways components Gli2 and Bmi-1 have been demonstrated to regulate stem cell self-renewal [77]. When injected into cleared mammary fat pads, Gli2 or Bmi-1 over expressing mammospheres were able to produce substantially more outgrowths than control mammospheres. Bmi-1 has been demonstrated to transcriptionally repress the p16^INK4a^ and p19^ARF^, suggesting a role for Bmi-1 in mammary stem cell cycle control. 

 Additional signaling pathways have been demonstrated to play important roles in stem cell quiescence, specifically the BMP pathway in skin. BMP and calcineurin signaling up-regulate the transcription factor NFAT1c that has been found to highly colocalize with CD34^+^ cells in the hair follicle [78]. NFAT1c represses transcription of CDK4, stalling cells in G_1_/S phase and maintaining quiescence. Loss of NFAT1c permits entry into the cell cycle, shortening telogen and prompting aberrant entry into anagen. 

While significant advances are being made in understanding quiescence control in normal adult stem cell populations, much less is known about control of quiescent CSC populations. Very few studies have been conducted specifically addressing control of quiescent CSCs, most likely due to the difficulty of isolating and analyzing pure CSC populations. If CSCs are truly derived from adult stem cells, then it is possible that Hes1, p21, p16^INK4a^, Rb family members, Bmi-1, and NFAT1c play significant roles in CSC regulation. Although rare, there are clues that at least some of these regulators are important in CSCs. In the colon cancer cell line HCT116, p21 null cells were found to produce tenfold smaller tumors in growth assays when compared to normal cells expressing p21 [79]. Under sphere forming conditions, p21 null cells were unable to form spheres, ceased proliferation, and eventually died. This p21 dependence was found to be associated with lack of E-cadherin expression and suppression of apoptosis signals, suggesting a more complex role for p21 in tumor cells than simply regulating cell cycle. Small molecule targeting of p21 or downstream p21 targets may therefore prove to be an effective means of forcing quiescent CSCs to cycle or undergo apoptosis. Cycling CSCs would be susceptible to chemotherapy and hopefully eliminated.

 Cancers frequently have aberrant signaling in the Wnt, Hedgehog (Hh), and Notch self-renewal pathways that likely contribute to cell cycle control and differentiation. Increased expression of Hes1 has been observed in ovarian, breast, and nonsmall cell lung carcinomas, suggesting active regulation of Notch signaling [36]. In melanoma, the slow cycling cells identified by Rosech et al., repress notch signaling directly though JARID1B interaction with the notch ligand Jagged 1 promoter, consequently reducing intracellular Notch and controlling proliferation [54]. Hes1 and Jagged1 may therefore be potential targets in future cancer treatments designed to target CSCs. Targeted reduction of Hes1 would increase Notch signaling, driving CSCs to proliferate and exhaust their proliferative potential, and making them more susceptible to conventional therapy. 

In colon cancers, mutations in APC or *β*-catenin are considered to be a driving force behind transformation [6]. In the presence of Wnt signal, *β*-catenin is no longer taken up by an APC-dependent degradation complex and translocates to the nucleus where it binds TCF/LEF transcriptions factors to control expression of cell cycle target genes. Loss of APC in crypt Lrg5^+^ cells has been demonstrated to be an important step towards initiation of intestinal adenomas [80]. Interestingly, cells expressing high Wnt downstream transcription factors TCF/LEF in primary sphere cultures demonstrated increased clonogenicity and the generation of both cycling and noncycling cells [81]. In tumors, these high Wnt expressing cells were located near stromal fibroblasts that provided signals to activate *β*-catenin-dependent transcription. This data suggests that CSC cell cycle control may not be entirely cell autonomous and partially regulated by microenvironmental signals. Targeting Wnt pathway regulators or the ability for CSCs to communicate with their stromal environment may represent potential mechanism for limiting CSC expansion and contribution to recurrence. 

There is also mounting evidence for the requirement of Hedgehog signaling in proliferation and survival of both colon and breast tumors. Active Hh-Gli signaling was found to contribute to the subpopulation of human colon CD133^+^ cells that were able to survive and self-renew in xenograft studies. In breast tumor CD44^+^/Cd24^−^ cells, the Hh pathway proteins Patch (PTCH1), Gli1, Gli2, and Bmi-1 all demonstrated increased expression over bulk tumors cells [77]. Like their adult mammary stem cell counterparts, overexpression of Bmi-1 in mammary CSCs suggests a potential role for p16^INK4a^ and p19^ARF^ in cell cycle regulation and suggests a potential drug target for improved CSC eradication.

While p21, p16, Notch, Wnt, and Hedgehog signaling may provide tempting targets for the removal of CSCs, targeting of these pathways would require meticulous targeting of CSC or titration of inhibitors to act on CSCs but not normal stem cell populations. Such treatments could severely weaken patients. Additionally, improper application of cell cycle inhibitors like p21 may fuel tumor growth and aggressiveness. The CDK inhibitor p21 acts as a tumor suppressor in dividing cells by protecting against genome instability and working with other tumor suppressors to subdue oncogenes [82, 83]. Loss of p21 combined with chemical induction of carcinogenesis has demonstrated increased induction of tumors and increased aggressiveness in resulting tumors [84, 85]. Combining widespread targeting of p21 with chemotherapy may have similar effects of tumors. These data highlight the necessity to be able to selectively target CSCs when using CDK inhibitors and add to the challenges ahead in developing treatments to better eradiate CSCs.

## 6. Conclusions and Future Directions

The limited data available on the regulation of quiescence equates to a poor understanding for the role of quiescence in tumor progression and recurrence. Exactly how and to what extent quiescence plays a role in tumor recurrence is at present unclear. What little evidence there is suggests that quiescence might be an important factor in tumor cell survival after conventional therapy. Mechanistically, CSC quiescence suggests an inherent means of resistance that when coupled with increased DNA repair or metabolic activity could explain the patterns of recurrence and acquired resistance currently observed in posttherapy cancer patients. The functionally relevant identification of quiescent CSCs though label-retaining assays may prove to be an important tool in ongoing CSC research.

Future research must focus on better understanding and targeting of quiescent CSC populations, specifically identifying regulators and factors that separate CSCs from normal stem cells. General targeting of p21, Bmi-1, Hes1, and other commonly shared cell cycle regulators might prove disastrous for patients if these treatments eradicate normal stem cell populations as well as CSCs. Aberrant regulation of normal stem cell characteristics presents a difficult paradox in fighting CSCs: how to target the cancer without harming normal stem cells. Hope exists that careful study of CSCs will identify new or differentially expressed targets that will specifically affect tumors, minimizing toxic side effects and leaving patients cancer free.

## Figures and Tables

**Table 1:** Comparison of characteritics between adult and cancer stem cells.

Characteristics	Adult Stem Cells	Cancer Stem Cells
Replicative Potential	Extensive proliferative capacity with the potential to exhaust regenerative ability	Extensive proliferative capacity with the potential to exhaust regenerative ability
Differentiation Ability	All lineages of the specific tissue	All heterogeneous lineages within the original tumor
Metabolic Activity	Low	Unknown
Signaling Pathway	Hedghog, Wnt, Notch, and BMP	Aberrant regulation of Hedghog, Wnt, Notch, BMP, and others
Cell Cycling Regulation	Slow cycling, tightly controlled	Potentially slow cycling, unknown
Location	Niche: Compartmentalized or associated with stromal layer	Unknown
Adhesion	Tightly Adhesive	Unknown
Migration Potential	No/Slow Migration	Epithelial to Mesenchymal Characteristics
